# Supplementary figures and images for: Titration-WB: A methodology for accurate quantitative protein determination overcoming reproducibility errors
Source: PLoS One. 2025 Jun 12;20(6):e0325052. doi: 10.1371/journal.pone.0325052 (PMC12161570; doi:10.1371/journal.pone.0325052)

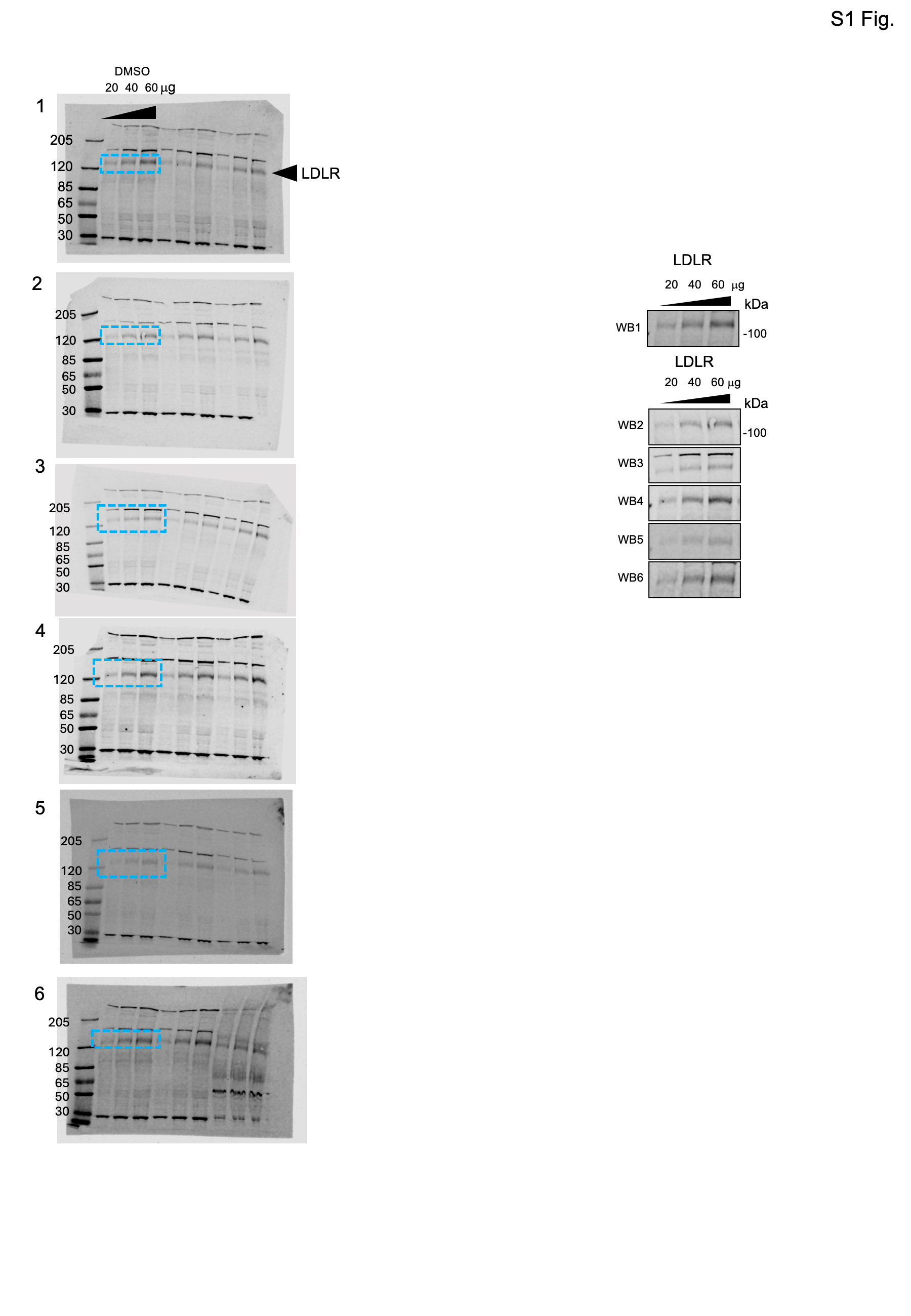

Supplement: S1 Fig — (TIF) [file pone.0325052.s001.tif]

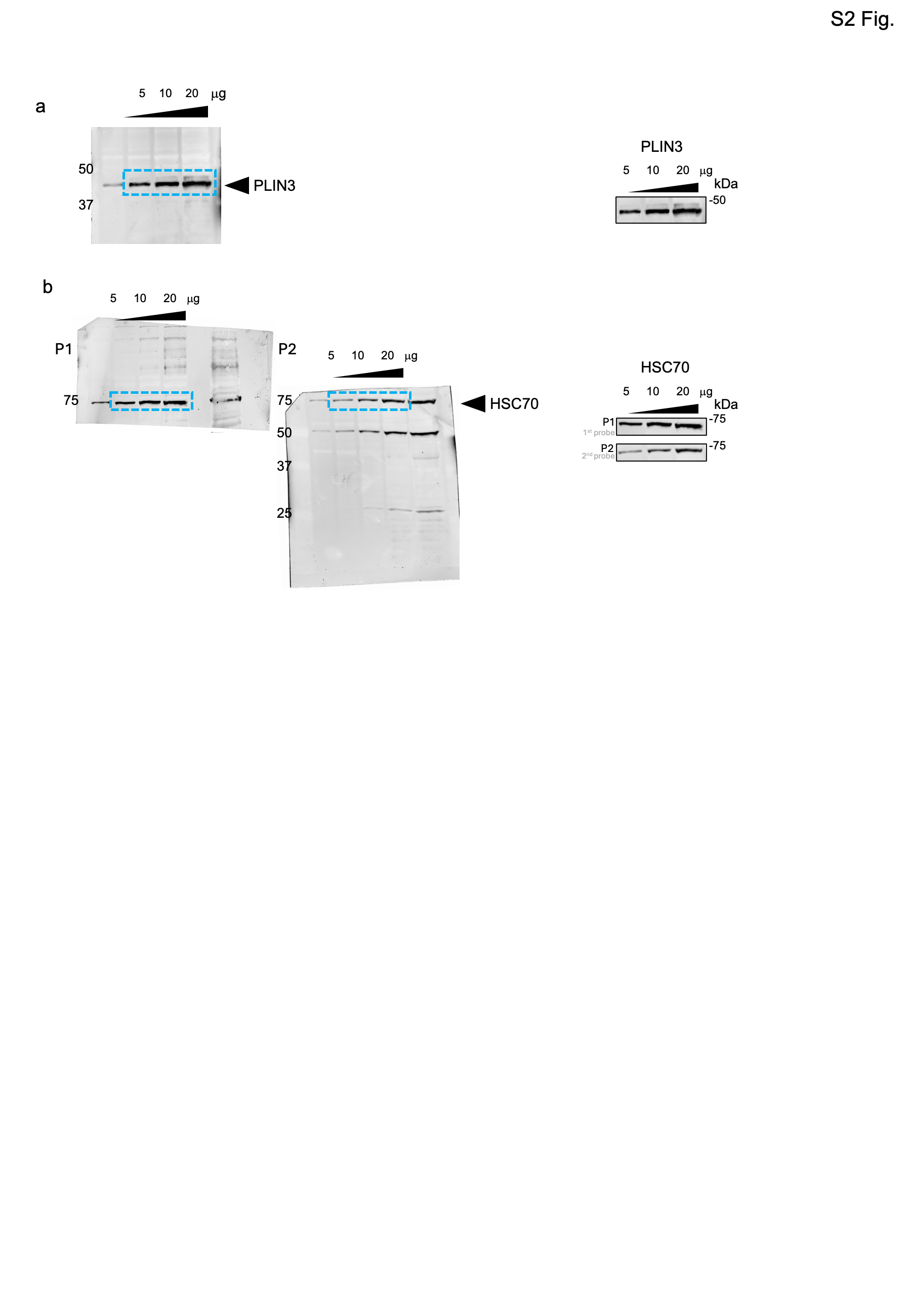

Supplement: S2 Fig — (TIF) [file pone.0325052.s002.tif]

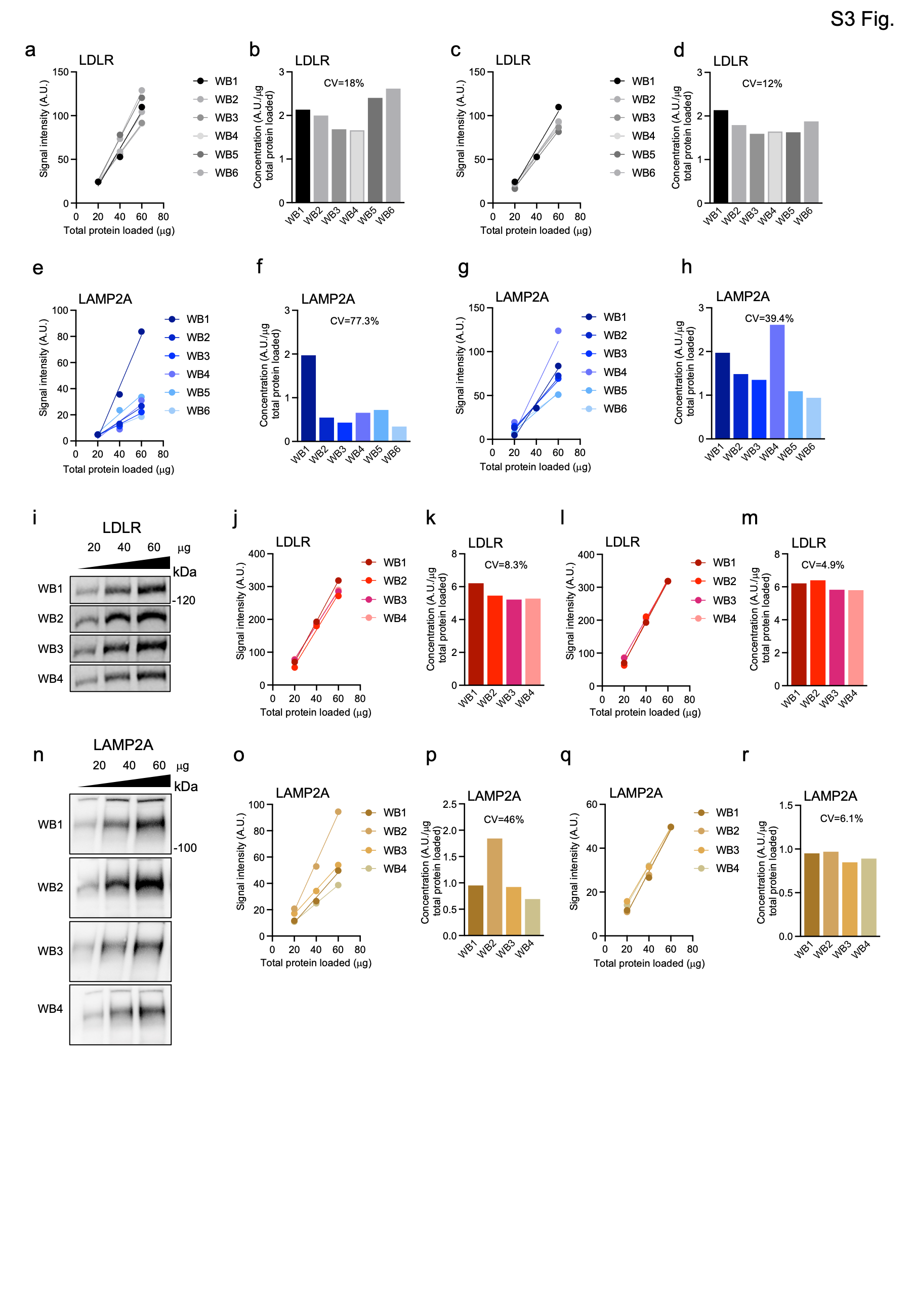

Supplement: S3 Fig — a) t-WB plots of LDLR after normalization of all points for WB1 20 μg point. b) LDLR concentrations from each WB expressed as signal intensity units (AU)/total protein mass (μg) at 20 μg and CV of the concentrations. c) t-WB plots of LDLR after normalization of all points for WB1 40 μg point. d) LDLR concentrations from each WB expressed as signal intensity units (AU)/total protein mass (μg) after normalization at 40 μg and CV of the concentrations. e) t-WB plots of LAMP2A after normalization of all points for WB1 20 μg point. f) LAMP2A concentrations from each WB expressed as signal intensity units (AU)/total protein mass (μg) after normalization at 20 μg and CV of the concentrations. g) t-WB plots of LAMP2A after normalization of all points for WB1 40 μg point. h) LAMP2A concentrations from each WB expressed as signal intensity units (AU)/total protein mass (μg) after normalization at 40 μg. i) Immunoblots of a single lysate of HepG2 cells treated by atorvastatin (5 mM), loaded at 20, 40, 60 μg of total protein onto 4 different WB membranes and probed for the LDLR. j) t-WB plots for LDLR for each WB1–4. k) LDLR concentrations from each WB expressed as signal intensity units (AU)/total protein mass (μg) and CV of the concentrations. l) t-WB plots of LDLR after normalization of all points for WB1 60 μg point. m) LDLR concentrations from each WB expressed as signal intensity units (AU)/total protein mass (μg) after normalization at 60 μg. n) Immunoblots the same 4 WB membranes shown in (i) with LAMP2A antibody. o) t-WB plots for LAMP2A for each WB1–4. p) LAMP2A concentrations from each WB expressed as signal intensity units (AU)/total protein mass (μg) and the overall CV. q) t-WB plots for LAMP2A after normalization of all data points using the WB1–60 μg data point. r) LAMP2A concentrations from each WB expressed as signal intensity units (AU)/total protein mass (μg) and the overall CV. (TIF) [file pone.0325052.s003.tif]

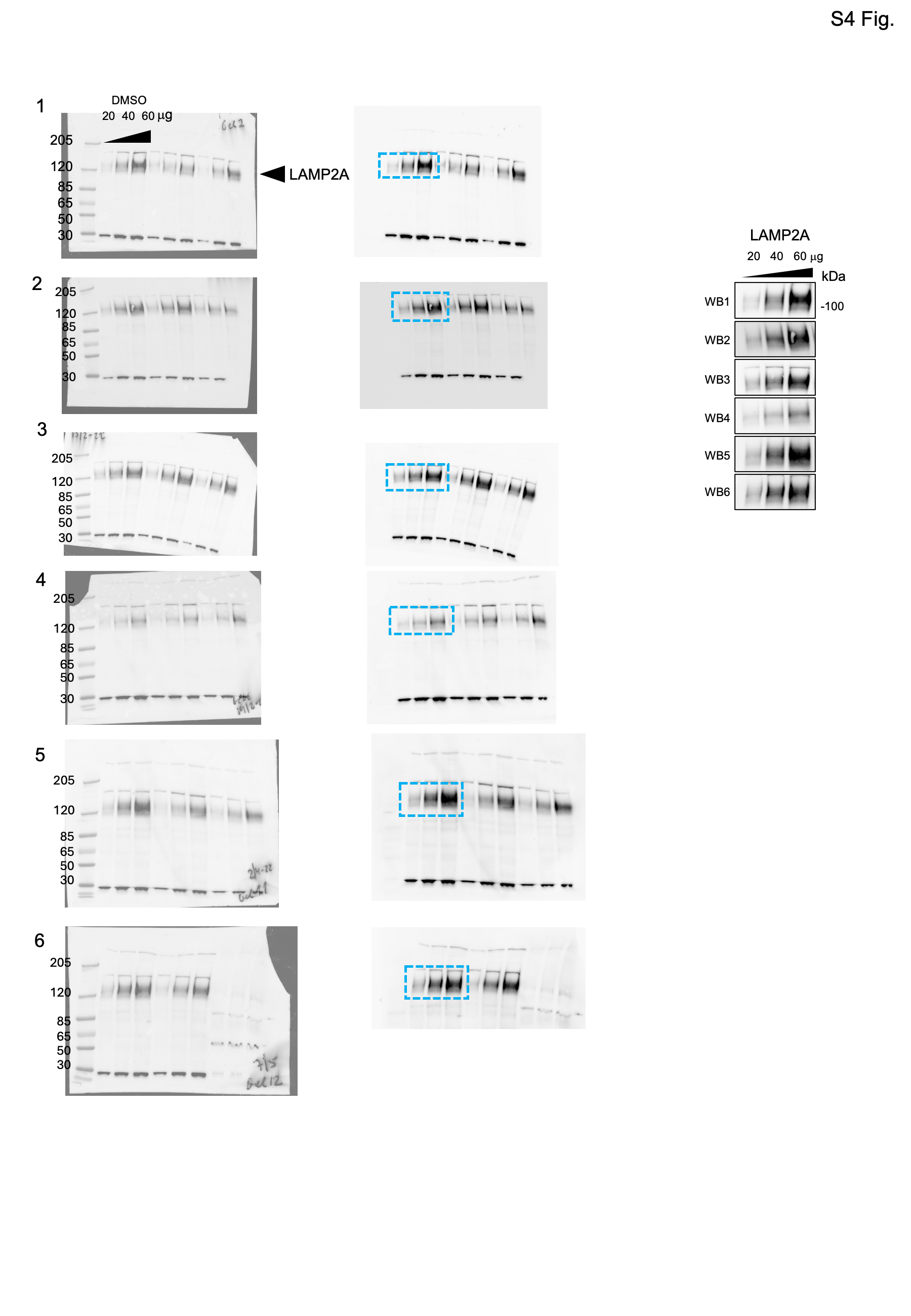

Supplement: S4 Fig — The blots are shown as composite image (left) and chemiluminescence only (right). (TIF) [file pone.0325052.s004.tif]

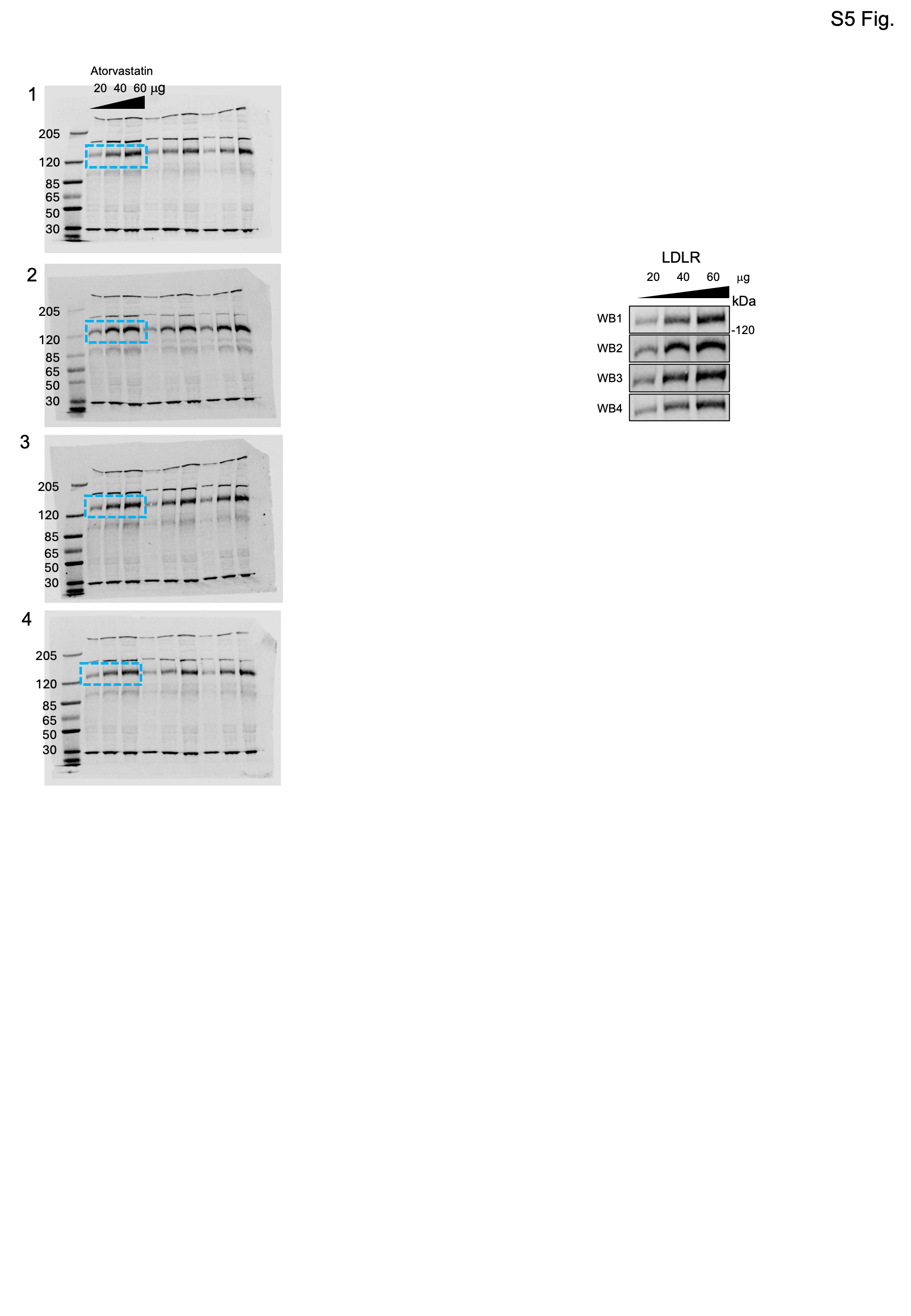

Supplement: S5 Fig — (TIF) [file pone.0325052.s005.tif]

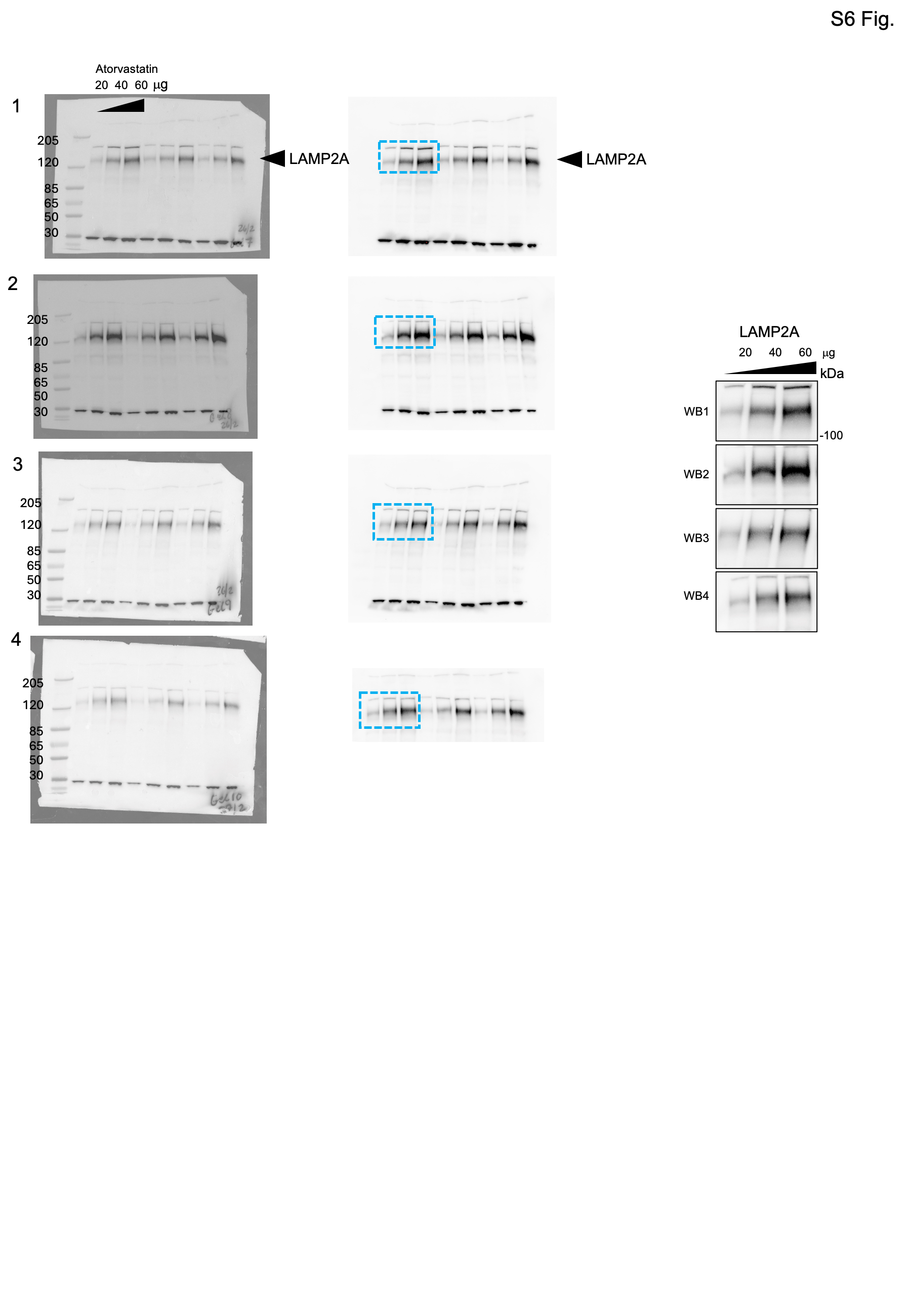

Supplement: S6 Fig — Original blots for S4 Fig n. The blots are shown as composite image (left) and chemiluminescence only (right). (TIF) [file pone.0325052.s006.tif]

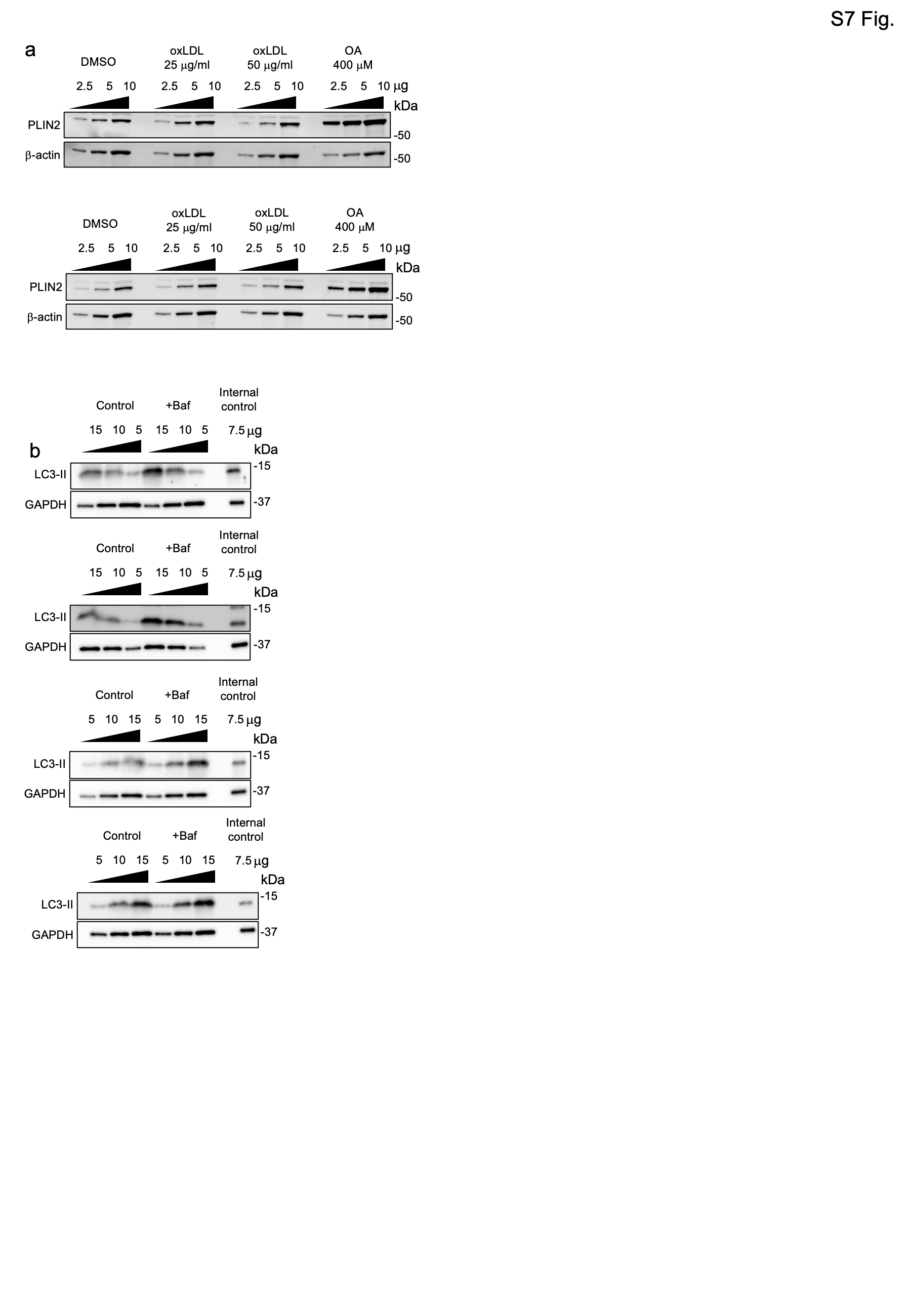

Supplement: S7 Fig — a) Immunoblots of PLIN2 and β-actin of THP-1 cells treated with vehicle (DMSO), 25 μg/ml oxLDL, 50 μg/ml oxLDL or 400 μM OA. Each cell lysate sample was loaded at 2.5, 5, 10 μg of total protein per well. Experimental repeats of Fig 3a (n = 3). b) Immunoblots of LC3-II and GAPDH of THP-1 cells treated with vehicle (DMSO) or Bafilomycin (+Baf). Each cell lysate sample was loaded at 5, 10, 15 μg of total protein per well. For each WB, a common internal control of THP-1 cells was loaded at 7.5 μg. Experimental repeats of Fig 3g (n = 5). (TIF) [file pone.0325052.s007.tif]

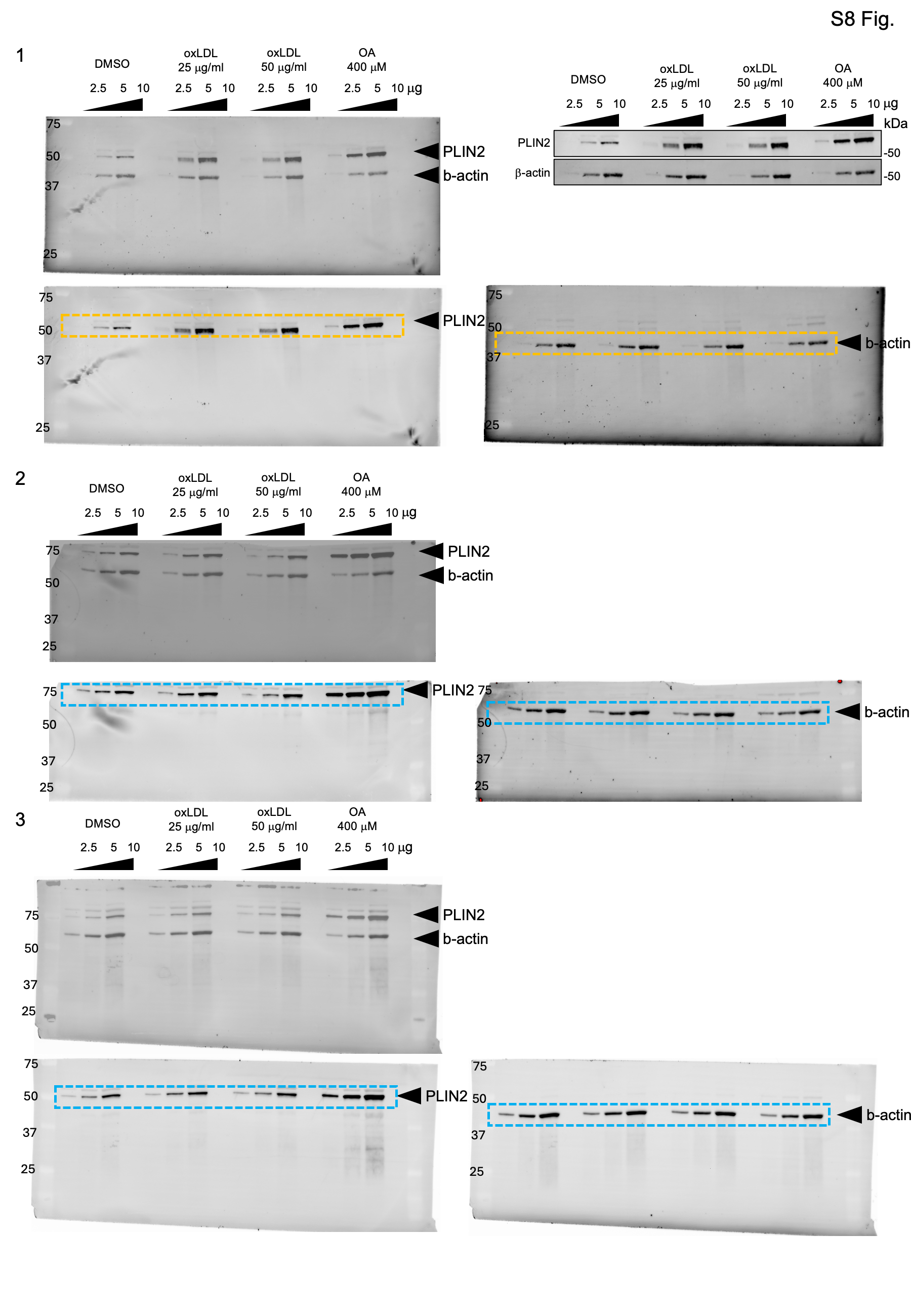

Supplement: S8 Fig — The blots are shown as composite image (top left) and fluorescence channels only (bottom left right for PLIN2 and bottom right for β-actin). The representative blots are highlighted in yellow, whereas the repeats are shown in blue. (TIF) [file pone.0325052.s008.tif]

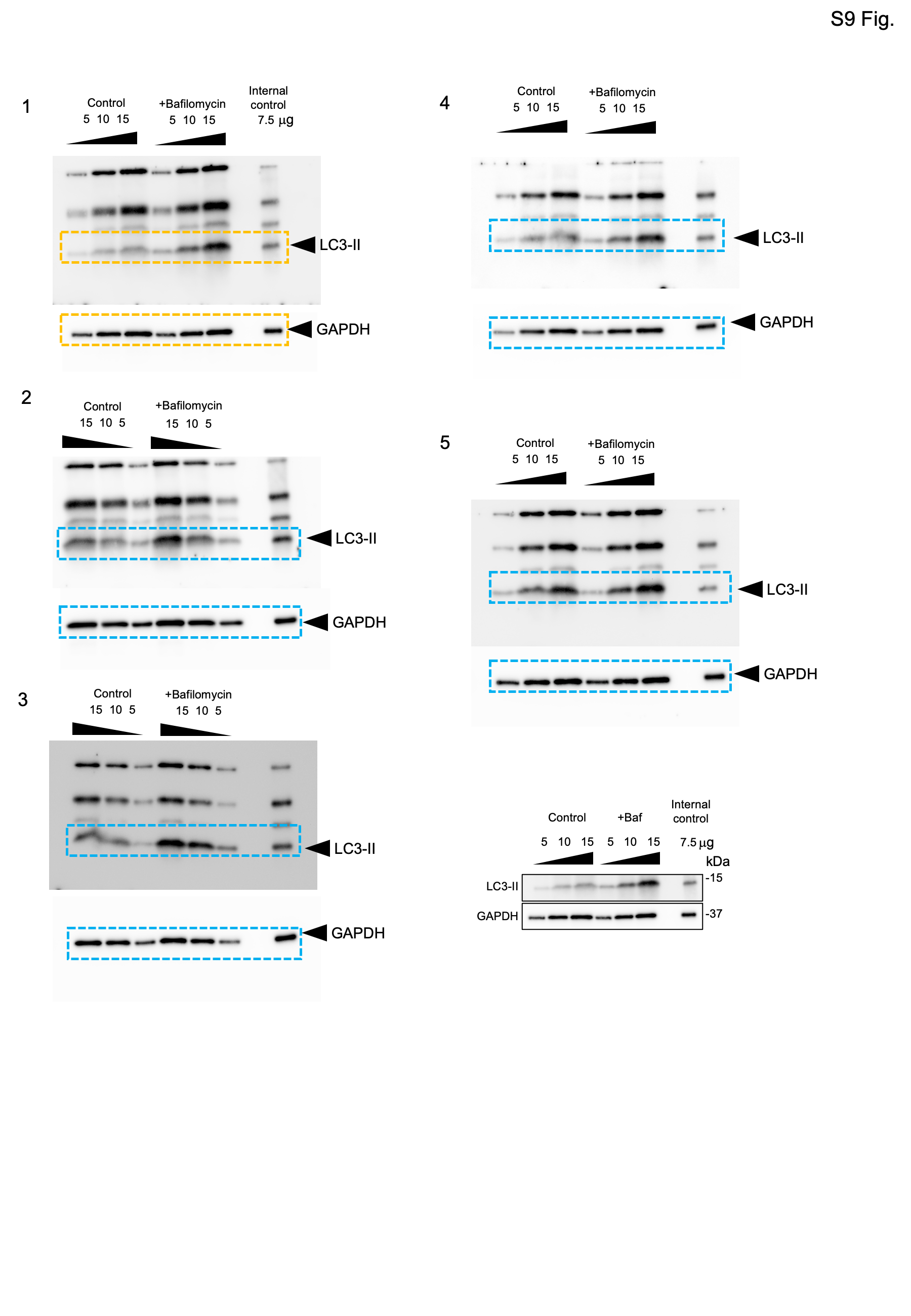

Supplement: S9 Fig — The blots are shown as chemiluminescence channels only (top for LC3-II and bottom for GAPDH). The representative blots are highlighted in yellow, whereas the repeats are shown in blue. (TIF) [file pone.0325052.s009.tif]
