# Supplementary material for: Titration-WB: A methodology for accurate quantitative protein determination overcoming reproducibility errors
Source: PLoS One. 2025 Jun 12;20(6):e0325052. doi: 10.1371/journal.pone.0325052 (PMC12161570; doi:10.1371/journal.pone.0325052)
Supplement: Attachment — Submitted filename: Rebuttal PONE-D-24-33858.pdf [file pone.0325052.s017.pdf]

Response to Reviewers comments for manuscript ID: PONE-D-24-33858 "**Titration-WB: A methodology for accurate quantitative protein determination overcoming reproducibility errors**" by Alice Maestri *et al.*

Original reviewer comments are shown in blue text, responses in black text. New text added to the revised manuscript based on Reviewer comments is shown with in red.

Reviewer #1:

This paper presents a valuable Titration-WB method that integrates Western blot with a titration assay to enhance protein quantification by reducing errors and enabling consistent inter-assay comparisons. While the approach significantly improves the accuracy and reliability of protein measurement, there are a few concerns that the authors need to address. The paper is suitable for acceptance after a major revision.

Answer: We thank the reviewer for recognising the significant improvement of our method on protein measurements.

1. The novelty of the paper needs further clarification, as the use of an internal standard is a well-established method applied in many analytical techniques, such as following article:
"Quantitative 31P NMR Spectroscopy Platform Method for the Assay of Oligonucleotides as Pure Drug Substances and in Drug Product Formulations Using the Internal Standard Method"
"A temperature-independent model of dual calibration standards for onsite and point-of-care quantification analyses via electrophoresis titration chip."
The authors should explicitly define the novel aspects of their work. While they claim that titration is the primary innovation, it appears that the method employed may simply involve dilution to create an internal standard. If this is the case, the term "dilution" might be more appropriate than "titration" for this context, as it seems to have limited relevance to titration itself.
The authors should refer to more recent work, especially within three years, now the references include a high proportion of outdated literature.

Answer: We thank the reviewer for this comment. We have realised that we have not extensively explained the core novelty of this method. t-WB employs the titration assay utilised in chemistry to generate a quantitative measurement of a protein of interest in a protein lysate. We have shown that this quantification is accurate and free from changes in antibody affinity and avidity towards the protein of interest and signal background. Importantly, t-WB protein quantification does not rely on housekeeping protein normalization, which is a source of variability and error. When not employed properly, the system is able to display errors in single pipetting and saturation.
With our examples of internal control normalizations, we have showcased that even though blot to blot variability of the same sample for a given target protein (notoriously known to be challenging to detect such as LDLR and LAMP2A) is high, the t-WB can be normalised and can give consistent results. The t-WB is thereby not a system based on internal standard calibration. We hope that with this updated manuscript and newly added experiments, we were able to clarify the method, its novelty and its applications.
Accordingly, we have cited more recent literature.

2. Some details in the paper, such as the explanation of R^2, are unnecessary. While R^2 is undoubtedly important, it is a well-known concept that can be found in textbooks, so there is no need to dedicate extensive text to introducing it.

Answer: We convene with the reviewer and we have reduced the explanation of the R^2 and kept it relevant only for the t-WB analysis.

3. The formatting of the paper is not consistent. For example, there is extra space before a paragraph on page 14. Additionally, the lack of line numbers makes it difficult for reviewers to reference specific positions in the text. I recommend standardizing the formatting and adding line numbers to improve readability and facilitate the review process.

Answer: We apologise for the lack of consistency throughout the manuscript. We have reformatted and added line numbers to the manuscript to aid the reviewing process.

4. There is a lot of empty space in the figures, giving the impression that some content is missing. The authors could consider adding an additional subplot from suppl Figures or removing two subplots to fill the space and enrich the figure, which would be suitable for the publication. Figures 1, 2, 4, and 5 should be formatted to match the style of Figure 3. The authors should refine the images and optimize the spatial layout to make them more suitable for publication.

Answer: We have modified the content of the figures, and we hope that the updated spatial layout will make the manuscript suitable for publication.

5. It's unclear why all points in the plot do not include error bars to indicate the relative standard deviation (RSD) range, only indicating CV of slopes. The current plot seems to only show single measurement results from one experiment. Including error bars would provide a clearer representation of variability and the reliability of the data.

Answer: We thank the reviewer for this comment. As a method paper, we have convened that a single experiment was sufficient to showcase the t-WB application. We have now integrated our result sections with additional experiments, both technical and experimental repeats.
For the LDLR and LAMP2A analysis, 6 independent WBs were performed with a control cell lysate (n_{exp}=1, n_{tech}=6) and 4 independent WBs were performed with a cell lysate treated with atorvastatin (n_{exp}=1, n_{tech}=4). Each technical repeat is shown individually both in the t-WB plot and in the bar graphs in Fig. 2 and Suppl. Fig. 1. All immunoblots have been submitted for reviewers' scrutiny in the file "Extended Data".
For the PLIN2 analysis 3 independent WBs were performed with 3 independent cell lysates (n_{exp}=3). The resulting data is shown combined in Fig. 3b-f. Each experimental repeat is shown in the new Suppl. Fig. 2a and the raw data is described in Suppl. Table 5.
For the LC3-II analysis, 5 independent WBs were performed with 5 independent cell lysates (n_{exp}=5). The resulting data is shown individually in Fig. 3h-o. Each experimental repeat is shown in the new Suppl. Fig. 2b and the raw data is described in Suppl. Table 6,7.

For most experiments, we preferred to visualise the individual WB runs instead of combining conditions, to showcase CV prior and after normalization.

6. If dilution range needs to be carefully optimized in page 3, then the authors should clearly

specify the detection range and conditions used in their experiments. This information is essential for understanding the accuracy and applicability of the method.

Answer: We thank the reviewer for this comment. As already mentioned in the manuscript, the detection range (total protein range) and conditions are shown in the figures and described in the methods section. We have described and referenced other publications that show how to determine a suitable detection range for WB. We thus believe that this should be standard procedure in all laboratories, and beyond the aim of the work presented in this manuscript.

7. some real picture is a little blurred (e.g. figure 3A, 3F), and there is inconsistent background brightness, running in different light intensity? like, WB1-7 in figure 3A, Gel 1 and Gel 2 in figure 5; WB1 and WB2-5 in figure S1R. If so, the author should state the background condition. Figure S1 included too many subfigures, splitting them to several parts is a better choice.

Answer: We apologise for image inconsistencies. After reassessing the quality of the images, we have excluded one membrane altogether for the WB1-7 (now Fig. 2) and WB1-5 in Suppl. Fig. 1. In regards to the crowdiness of Supplementary Figure 1, we have reduced the panels only to show the normalizations at 60 µg.
For clarity, we have included all western blot images in the file "Extended Data" and added a Suppl. Fig. 2 with the experimental immunoblot repeats of Fig. 3.

8. No conclusion; I recommend that the authors separate the Conclusion from the Discussion to clearly present the key contributions of this work.

Answer: We thank the reviewer for their input. We have now added a conclusion section as shown below:

Lines 345-364:

"Conclusion:
The titration-Western Blot method represents a significant advancement in the field of protein quantification, addressing long-standing challenges associated with traditional Western blotting. By integrating principles of titration analysis, t-WB offers a robust framework for accurate and reproducible quantitative protein measurements, eliminating reliance on housekeeping proteins and mitigating biases introduced by experimental variability. Through the use of serial dilutions and regression analysis, t-WB enables precise quantification while providing additional quality control metrics, such as the R^2 values and the intercepts, to identify and correct eventual experimental inconsistencies.
This method's versatility and reliability have been validated across various experimental conditions, demonstrating its capability to reduce inter-experiment variability, improve cross-blot comparisons, and uncover subtle biological changes often obscured by conventional normalization strategies. By overcoming limitations inherent to traditional WB protocols, t-WB sets a new standard for quantitative protein analysis, offering researchers a powerful tool to achieve greater accuracy and reproducibility in their studies.
As experimental methodologies continue to evolve, t-WB serves as a foundation for further innovation in protein quantification, bridging the gap between qualitative detection and precise quantitative analysis. Its adoption is poised to enhance the rigor and reproducibility of

molecular biology research, ensuring more reliable insights into the complex dynamics of protein expression and regulation."

9. The reference formatting needs to be standardized, including consistent use of journal name abbreviations.

Answer: We have reformatted the references according to PlosOne guidelines.

Reviewer #2:

1. The authors should describe the "titration assay" more in the Abstract and Introduction sections, and it is also not clear what the advantage of merging it and WB for quantifying proteins is.

Answer: We thank the reviewer for this comment, and we have now included a brief introduction of the titration assay in both the abstract and introduction as follow:

Lines 38-53:
Abstract:
"Western blotting (WB) is a cornerstone technique for protein detection and quantification in molecular biology. However, its semi-quantitative nature, reliance on housekeeping protein normalization, and susceptibility to technical variability often undermine data accuracy and reproducibility. To address these limitations, we introduce titration-based Western blotting (t-WB), as innovative quantitative approach that uses serial dilutions of protein samples to generate regression curves for precise protein quantification. The method mitigates common errors, including loading inaccuracies and signal saturation, by leveraging the R^2 value as a quality control metric and calculating the regression line. Its slope is then used as a measure of protein concentration, expressed as signal intensity/total protein loaded. The advantage of t-WB is the removal of housekeeping protein normalization, eliminating thus the bias created by experimental conditions that may alter housekeeping protein levels. t-WB was validated across diverse setups, demonstrating its robustness in minimizing inter-experiment variability and improving accuracy by normalizing to a single internal control. By standardizing workflows, t-WB ensures reproducibility, uncovers subtle biological changes, and resolves biases inherent to classical WB protocols."

Lines 88-98:
Introduction:
"To address these limitations, we introduce the t-WB method, which combines part of the principles of classical titration assays used in chemistry, with the WB technique. Titration assays involve systematically varying the concentration of a substance of interest and measuring the subsequent change in signal output intensity to determine the concentration of the analyte. In t-WB, we expand this concept by generating a function that relates the signal intensity of the protein of interest to the amount of total protein loaded onto the gel at multiple concentrations. The first-order derivative of this function is then used to quantitatively estimate the target protein concentration (9). While not offering absolute quantification of protein concentration, which would require standards such as recombinant proteins of known concentrations, t-WB still provides significant improvements in accuracy and precision over classical WB techniques."

2. In this study, the total protein content was quantified by the Lowry method. When authors loaded the samples according to the quantification results, the errors described here, which

were not expressed by a fold change, also could largely come from the quantification method. These errors could stem from a variety of sources, including variations in sample preparation, instrument calibration, or even human error. It's important to understand these potential sources of error to ensure the accuracy of our results.

Answer: We convene with the reviewer that there are many factors contributing to the variability of results. We have added a practical example of how these major errors are detectable in our system in the new Fig. 1. We are confident that the examples shown after Fig. 1 and their variabilities are not due to these aforementioned errors.

3. By using WB to compare the expression patterns of the interested proteins, we usually use internal control proteins such as GAPDH or a-tubulin to eliminate the errors from the dye-based quantification results. A similar idea is also presented in Fig.5. However, it is already a typical and regular process done by most of the labs, which makes the idea in this study lack novelty and improvement.

Answer: We agree with the reviewer that normalization to a housekeeping protein is the standard procedure for most laboratories when it comes to WB analysis. As explained, this is indeed one of the reasons for the too-frequent lack of reproducibility of results. A main reason for the development of the t-WB pipeline is to exclude the use of housekeeping protein, which is a great source of bias and as carefully showed and discussed in our work. In Fig. 3, we show the t-WB analysis of PLIN2 and LC3-II and the comparisons with their respective estimations by performing the classical WB in which we use β-actin and GAPDH - PLIN2/β-actin and LC3-II/GAPDH, respectively. In the first example, β-actin shows a treatment-dependent variability which causes a gross overestimation of PLIN2 in the oleic acid (OA)-treated sample. In the second, GAPDH does not have such variability, but it still minimises the bafilomycin-induced LC3-II increase at lower total protein loaded. With the t-WB analysis we obtain a more reliable result, as it is independent of the housekeeping protein. When we perform the t-WB in our laboratory, we do not collect data related to housekeeping proteins. However, for the purpose of showcasing the method, we have included these data.
